# Cyclic compression emerged dual effects on the osteogenic and osteoclastic status of LPS-induced inflammatory human periodontal ligament cells according to loading force

**DOI:** 10.1186/s12903-019-0987-y

**Published:** 2020-01-06

**Authors:** Ru Jia, Yingjie Yi, Jie Liu, Dandan Pei, Bo Hu, Huanmeng Hao, Linyue Wu, Zhenzhen Wang, Xiao Luo, Yi Lu

**Affiliations:** 1 Key Laboratory of Shaanxi Province for Craniofacial Precision Medicine Research, College of Stomatology, Xi’an Jiaotong University, Xi’an, China; 2 Department of Prosthodontics, Stomatological Hospital, College of Medicine, Xi’an Jiaotong University, No. 98 Xiwu Road, Xi’an, 710004 Shaan Xi China; 3 Department of Physiology and Pathophysiology, Xi’an Jiaotong University Health Science Center, No. 76 Yanta West Road, Xi’an, 710061 Shaanxi China

**Keywords:** Periodontitis, hPDLCs, LPS, Dynamic loading, Osteogenic differentiation

## Abstract

**Background:**

Appropriate mechanical stimulation is essential for bone homeostasis in healthy periodontal tissues. While the osteogenesis and osteoclast differentiation of inflammatory periodontal ligament cells under different dynamic loading has not been yet clear. The aim of this study is to clarify the inflammatory, osteogenic and pro-osteoclastic effects of different cyclic stress loading on the inflammatory human periodontal ligament cells (hPDLCs).

**Methods:**

hPDLCs were isolated from healthy premolars and cultured in alpha minimum Eagle’s medium (α-MEM). Lipopolysaccharides (LPS) were used to induce the inflammation state of hPDLCs in vitro. Determination of LPS concentration for the model of inflammatory periodontium was based on MTT and genes expression analysis. Then the cyclic stress of 0, 0–50, 0–90 and 0–150 kPa was applied to the inflammatory hPDLCs for 5 days respectively. mRNA and protein levels of osteogenic, osteoclastic and inflammation-related markers were examined after the treatment.

**Results:**

MTT and RT-PCR results showed that 10 μg/ml LPS up-regulated *TNF-α, IL-1β, IL-6, IL-8* and *MCP-1* mRNA levels (*P* < 0.05) and did not affect the cell viability (*P* > 0.05). The excessive loading of stress (150 kPa) with or without LPS strongly increased the expression of inflammatory-related markers *TNF-α*, *IL-1β*, *IL-6*, *IL-8*, *MCP-1* (*P* < 0.05) and osteoclastic markers *RANKL*, *M-CSF*, *PTHLH* and *CTSK* compared with other groups (*P* < 0.05), but had no significant effect on osteogenic genes. While 0–90 kPa cyclic pressure could up-regulate the expression of osteogenic genes *ALP, COL-1*, *RUNX2, OCN, OPN* and *OSX* in the healthy hPDLSCs.

**Conclusions:**

Collectively, it could be concluded that 0–150 kPa was an excessive stress loading which accelerated both inflammatory and osteoclastic effects, while 0–90 kPa may be a positive factor for the osteogenic differentiation of hPDLCs in vitro*.*

## Background

Periodontitis is a chronic infective disease of the periodontium caused by bacteria. It especially occurs among the elderly, and may develop into the bone loss and dental deficiency, which is one of the severest consequences. To repair the missing teeth for these patients, the control of inflammation state and occlusal force on the involved teeth is the key point which should be well considered. However, there is no conclusion about the differences between periodontitis and healthy abutments under the dynamic mechanical stress, and the range of occlusal force that periodontitis teeth can bear. What’s more, the osteogenesis and osteoclast differentiation of the inflammatory periodontal ligament cells under different dynamic loading has also not been clear yet.

Endotoxin is an important toxic component in the occurrence and development of periodontitis. When human periodontal ligament cells (hPDLCs) were exposed to Lipopolysaccharides (LPS), the major active component of endotoxins, the expressions of pro-inflammatory cytokines was increased [1]. Tumor necrosis factor (TNF)-α, and interleukin (IL)-1β, − 6, − 8, − 10, − 11 etc. are the pro-inflammatory cytokines secreted to cause inflammatory response, loss of periodontium and alveolar bone, which would eventually lead to irreversible teeth loosening and falling off [2–4]. Kato reported that 1 and 10 μg/ml LPS could affect osteoblastic differentiation and up-regulate IL-1β, IL-6, and IL-8 production in human periodontal ligament stem cells (hPDLSCs) [5]. Besides, in the research of Liu, applying LPS on hPDLCs could trigger the inflammation reaction [6]. However, the concentration and duration of LPS treatment for modeling the periodontitis in hPDLCs in vitro preferably was remained to be clear and definite.

The periodontal ligament (PDL) which mainly contains fibroblasts is connecting the root and alveolar bone and responsible for the formation of collagen fiber networks. Meanwhile, a few osteoblast-like fibroblasts in PDL has the capacity to give rise to bone cells and cementoblasts [7]. Because of the components, PDLCs are able to bear physiological mastication. In recent studies, the mechanical loading within the physiological range has been found to stimulate the differentiation of PDLCs in vitro [8, 9]. Compression is the way that scholars simulate the stress state of periodontal ligament cells under normal occlusion. In PDLCs, 65 g/cm^2^ (245 kPa) static compression given by weight could take part in the initiation of osteoclastogenesis. It could up-regulate the expression of pro-osteoclastogenic cytokine, like receptor activator for nuclear factor-κB ligand (RANKL) and parathyroid hormone-related protein (PTHrP), and the pro-inflammation cytokines including IL-8 and IL-11 [10, 11]. It has been demonstrated that 150 psi (1034 kPa) static compression by air pressure could up-regulate the expressions of matrix metalloproteinases (MMPs)-1/7/9, which are the cytokines regulating the degradation of extracellular matrix, and inflammation-related genes in inflammatory hPDLCs [12]. However, there is no defined physiological pressure range for hPDLCs because of the different ways of pressure loading. As we known, in the process of mastication, the occlusal force borne by periodontium is discontinuous rather than unchangeable. Thus, it would be better to study the inflammation status and the osteogenic and osteoclast differentiation of the hPDLCs using a dynamic loading way to mimic the functional status. Therefore, in this present study, by applying cyclic air compression, we compared the expression differences of pro-inflammation, pro-osteoclastogenic and osteoblast-related cytokines between healthy and LPS-induced inflammatory hPDLCs under different dynamic loadings. This work may provide the foundation for clearing the reasonable force range of hPDLCs and give the reference for stress designing of the periodontitis abutment teeth in clinic.

## Methods

### Cell isolation and culture

In total, 12 healthy and noncarious premolars from three male and three female donors aged from 18 to 30 years old who had received orthodontic treatment at the College of Medicine & Hospital of Stomatology, Xi’an Jiaotong University (Xi’an, China) were obtained for hPDLCs. The primary cells were collected by scrapping the middle third of the roots, minced into pieces at about 1 mm^3^, and subjected to 0.3% collagenase type I (Sigma, USA) at 37 °C with gently shaking for 30 min. After centrifugation, the precipitate was transferred to culture flasks (Corning, USA) with α-minimum essential medium (a-MEM) (Hyclone, USA) supplemented with 10% fetal bovine serum (Gibco, USA), 100 U/ml penicillin and 100 mg/ml streptomycin (Sigma, USA), and then cultured in a humidified atmosphere of 5% CO_2_ at 37 °C. The teeth were collected with informed consent of the donors and the approval of the Ethics Committee of College of Medicine & Hospital of Stomatology, Xi’an Jiaotong University (approval number No. 2018–134). All the cells used in this study were at passage 4 after 1–2 weeks of culture.

### Immunocytochemistry staining

hPDLCs at passage 4 were seeded at a density of 1 × 10^4^/well and covered in advance with circular coverslips and incubated for 48 h at 37 °C. Cells were then rinsed and fixed with 4% paraformaldehyde at room temperature. Following a further wash, 0.25% Triton X-100 was added into the 24-well plates, which were incubated at 37 °C for 15 min. After incubated with 1% bovine serum albumin (Gibco, USA) and 22.52 mg/ml glycine in PBS + 0.1% Tween-20, hPDLCs were incubated with anti-vimentin (1:100; ab24525; Abcam, USA) and anti-cytokeratin (1:200; AM06387SU-N; OriGene Technologies, China) primary antibodies overnight at 4 °C. The immunohistochemistry assay kit (SP9001; OriGene Technologies; China) was used for immunocytochemical staining according to the manufacturer’s instructions, and a 3,3′-diaminobenzidine was used to stain positive cells. The cells were examined by an inverted microscope (FSX100; Olympus Corporation, Japan).

### Inflammation induction

hPDLCs were plated at a density of 2 × 10^3^ cells/well in 96-well plates and 5 × 10^5^ cells/well in 6-well plates. And then the hPDLCs were cultured in basal medium containing LPS (SMB00610, Sigma, USA) with concentration of 0, 0.1, 1.0, 10, 100 and 500 μg/ml for 24 h, 48 h and 72 h respectively.

### MTT assay

After treated with different dosage of LPS, the cell viability of hPDLCs was measured by 3-(4,5-dimethylthiazol-2-yl)-2,5-diphenyltetrazolium bromide (MTT) (Beyotime, China) assay. The MTT was added into the medium in 0.2 mg/ml for 4 h. Then the plates were centrifuged at 1000 rpm for 10 min and the supernatant was discarded. The crystal was dissolved with dimethyl sulfoxide (DMSO) (Sigma, USA) (200 μl/well), and the optical density of the solution was measured at 490 nm on an automatic imark Microplate Absorbance Reader (Bio-Rad Laboratories, USA).

### Application of dynamic cyclic stress

hPDLCs were plated at a density of 5 × 10^5^ cells/well in 6-well plates, and cultured in basal medium. A self-designed, hydraulic pressure-controlling cellular strain unit following the model developed by Yousefian et al. [13] was used here to simulate compressive stress on the cultured hPDLCs. This assembly was patented approval (No. CN201310254749) and the specific characteristics, operation and selection of pressure loading conditions of the pressure loading device was declared in the previous studies [14, 15]. After treated with or without LPS, all the plates were applied to cyclic hydrostatic pressure of 0, 0–50, 0–90 and 0–150 kPa (0.1 Hz) using this dynamic hydrostatic pressure booster, 1 h/day for 5 days. The medium was replaced every day.

### Quantitative real-time polymerase chain reaction (real-time PCR)

Total RNA was isolated from hPDLCs using TRIzol (TAKARA, Japan) and converted to cDNA by a commercial RT-PCR Kit according to the manufacturer’s instructions (TAKARA, Japan). RT-PCR was performed using SYBR Premix Ex Taq II (TAKARA, Japan) following a real-time PCR detection System (Applied Biosystems, USA). Expression data were normalized to the amount of β-actin mRNA using the –ΔΔCt method. The primers for all the genes are listed in Table 1. Each reaction was performed in triplicate.

**Table 1:** Primers used for quantitative real-time PCR analysis

Gene	Forward primer	Reverse primer
IL-1β	AACCTCTTCGAGGCACAAGG	AGATTCGTAGCTGGATGCCG
IL-6	AGTGAGGAACAAGCCAGAGC	AGCTGCGCAGAATGAGATGA
IL-8	ACTGAGAGTGATTGAGAGTGGAC	AACCCTCTGCACCCAGTTTTC
TNF-α	CTGCACTTTGGAGTGATCGG	TCTCTCAGCTCCACGCCATT
MCP-1	CAGCCAGATGCAATCAATGCC	TGGAATCCTGAACCCACTTCT
ALP	GGACCATTCCCACGTCTTCAC	CCTTGTAGCCAGGCCCATTG
COL-1	CCAGAAGAACTGGTACATCAGCAA	CGCCATACTCGAACTGGAATC
RUNX2	CCCGTGGCCTTCAAGGT	CGTTACCCGCCATGACAGTA
OCN	AATCCGGACTGTGACGAGTTG	CAGCAGAGCGACACCCTAGAC
OPN	GCCGAGGTGATAGTGTGGTT	ACTCCTCGCTTTCCATGTGT
OSX	CCTCTGCGGGACTCAACAAC	AGCCCATTAGTGCTTGTAAAGG
RANKL	GAAAGCAAATGGTGTGGCCG	ACGTCACATCCCTGGTACAC
M-CSF	CTA AGCTGGACGCACAGACCA	TCTCAGGCTGCACACCTT
PTHLH	GTGTCCCCTAACTCCAAGCC	TTGAGCGGCTGCTCTTTGTA
CTSK	GGGGGACATGACCAGTGAAG	CAGAGTCTGGGGCTCTACCT
β-actin	TGGCACCCAGCACAATGAA	CTAAGTCATAGTCCGCCTAGAAGCA

### Western blotting analysis

The total protein of hPDLCs in each group was extracted by Ripa lysate (Boster, China) according to the manufacturer’s instructions. And the protein concentration was determined using the bicinchoninic acid (BCA) protein assay kit (Thermo scientific, USA). About 20 μg of protein was separated with 10 and 15% SDS-PAGE gels. The isolated protein was transferred onto polyvinylidenedifluoride membrane, and then blocked with 5% non-fat dry milk in TBS containing 0.1% Tween for 1 h at room temperature. Then the membranes were trimmed to narrow strips based on the molecular weight of the target proteins according to the markers (#26616, Thermo Scientific, USA). These strips were probed with an antibody to a single target protein. The membranes were incubated with the appropriate primary antibodies overnight at 4 °C. After washing, the membranes were incubated with a secondary horseradish peroxidase (HRP)-coupled antibody and processed for an enhanced chemiluminescence detection using Immobilon HRP substrate (Millipore, USA). Signals were visualized and analyzed on a UVP Vision Works LS BioSpectrum (Aplegen, USA). The intensity of bands was quantified using IMAGEJ software (National Institute of Health, USA). The ratio of the intensity of the target protein to that of β-actin loading control was calculated to represent the expression level of the protein. Antibodies were as follows: anti-IL-1β (1:1000) (ab2105), anti-TNF-α (1:1000) (ab8348), anti- Collagen-1(COL-1) (1:2000) (ab90395), anti- runt-related transcription factor 2 (RUNX2) (1:2000) (ab23981), anti-RANKL (1: 1000) (ab9957), anti-β-actin (1: 1000) (ab8224) (Abcam, UK).

### Statistical analysis

Statistical analysis was performed by SPSS 19.0 software (IBM, USA), using one-way (repeated-measures) ANOVA for differences across experimental groups in conjunction with Tukey’s post hoc test to compare the differences between the treatment groups. Each experiment was performed three times. Data were expressed as means ± SEM. *P* values of < 0.05 were considered statistically significant.

## Results

### Cell characterization and LPS-induced inflammation of hPDLCs in vitro

**Fig. 1:**
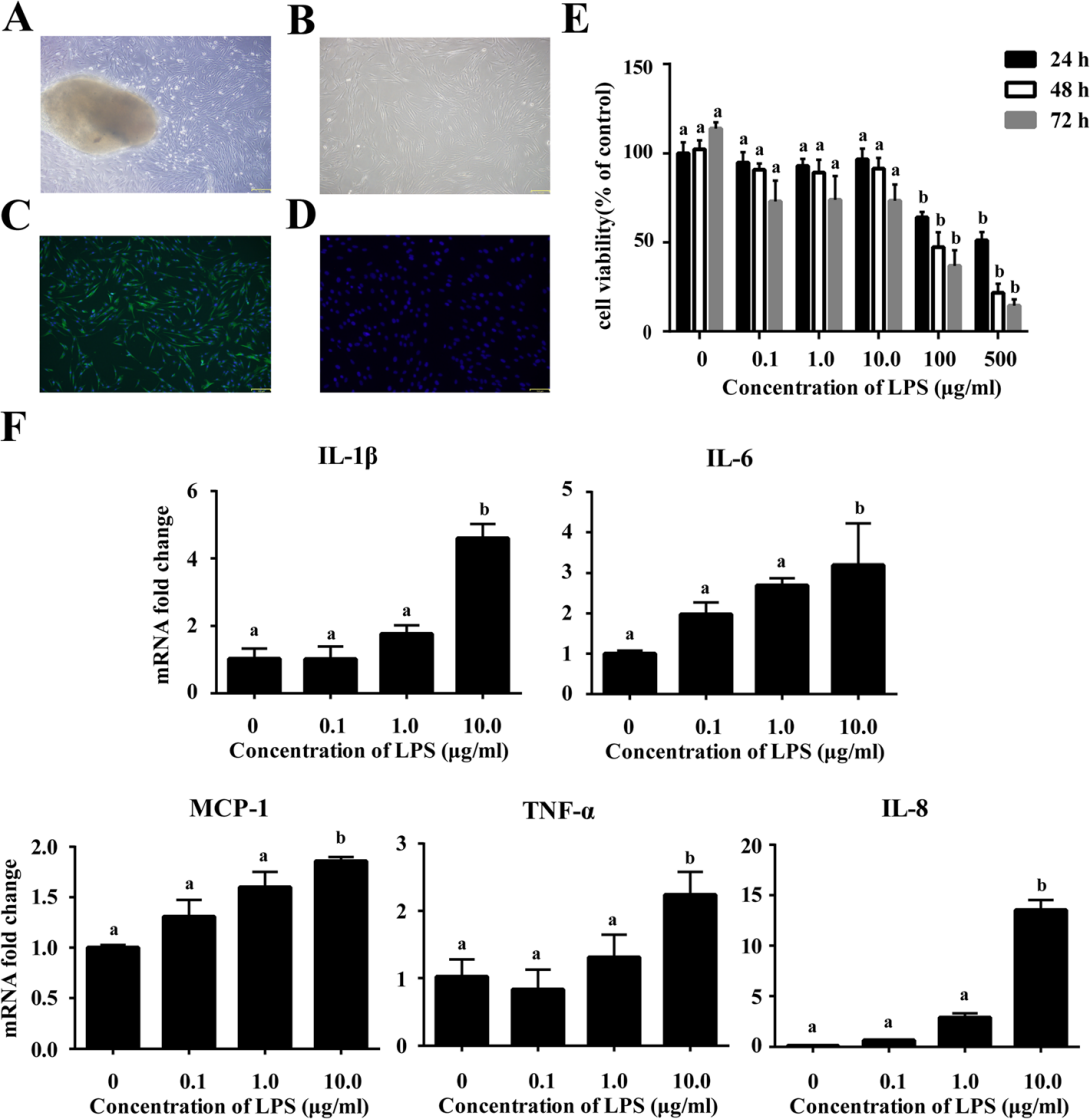
Cell characterization and LPS-induced inflammation of hPDLCs in vitro*.* Primary cells grew out from the tissue explants (**a**) and were spindle shaped (**b**). Immunocytochemistry staining showed that cells were vimentin positive (**c**) and cytokeratin negative (**d**) (magnification: 100×, scale bar: 150 μm). After treated by different concentrations of LPS (range = 0–500 μg/ml) for 24, 48 and 72 h respectively, cell viability of hPDLCs was evaluated with MTT assay (**e**). mRNA expressions of pro-inflammatory cytokines *IL-1β*, *IL-6*, *IL-8*, *MCP-1* and *TNF-α* in hPDLCs after 0.1, 1.0 and 10 μg/ml LPS treatment were detected using real-time PCR (**f**). Data were represented as means ± SEM, *n* = 6 (hPDLCs from six donors). The bars with different lowercase letters were significantly different from each other (*P* < 0.05), and those with the same letter exhibited no significant difference

hPDLCs grew out from the tissue explant after 7 and 10 days of culture (Fig. 1a). Spindle shapes were observed, and a number of cells were distributed in a circinate pattern with rapid proliferation (Fig. 1b). The cells were vimentin positive (Fig. 1c) and keratin negative (Fig. 1d) according to immunochemistry staining, indicating that these primary cells were of mesenchymal origin.

We then examined whether LPS induced the inflammation in hPDLCs without affecting cell viability. hPDLCs were exposed to increasing concentrations of LPS (at range = 0–500 μg/ml) for 24, 48 and 72 h and cell viability was determined. According to the results of the MTT assay, the proliferation of hPDLCs showed a significant reduction after treated with 100 or 500 μg/ml LPS for 24 h, 48 h and 72 h compared with the control group (*P* < 0.001). While the other experimental groups, which were treated with 0.1, 1.0 or 10 μg/ml LPS, showed no significant difference in cell proliferation and viability compared with the control group (*P* > 0.05) (Fig. 1e).

Since the high concentration (100 and 500 μg/ml) of LPS treatment would affect the bioactivity of hPDLCs which could be excluded for the modeling, we then investigated the inflammatory response of the hPDLCs under 0.1, 1.0, 10 μg/ml LPS treatment. The mRNA expressions of inflammatory cytokines were exhibited using real-time PCR. The results indicated that 10 μg/ml LPS induced the expression of pro-inflammatory cytokines, including *IL-1β*, *IL-6*, *IL-8*, monocyte chemotactic protein 1 (*MCP-1*) and *TNF-α* in hPDLCs, compared with the control group (*P* < 0.05). However, the mRNA expression of pro-inflammatory cytokines in those 0.1 and 1.0 μg/ml LPS groups showed no statistical significant up-regulation compared to the control group (*P* > 0.05) (Fig. 1f). According to the results of this section, 10 μg/ml would be chosen as the working concentration of LPS for the inflammation induction model of hPDLCs in vitro.

### The pro-inflammatory effects of different dynamic cyclic stress on LPS-induced inflammatory hPDLCs

**Fig. 2:**
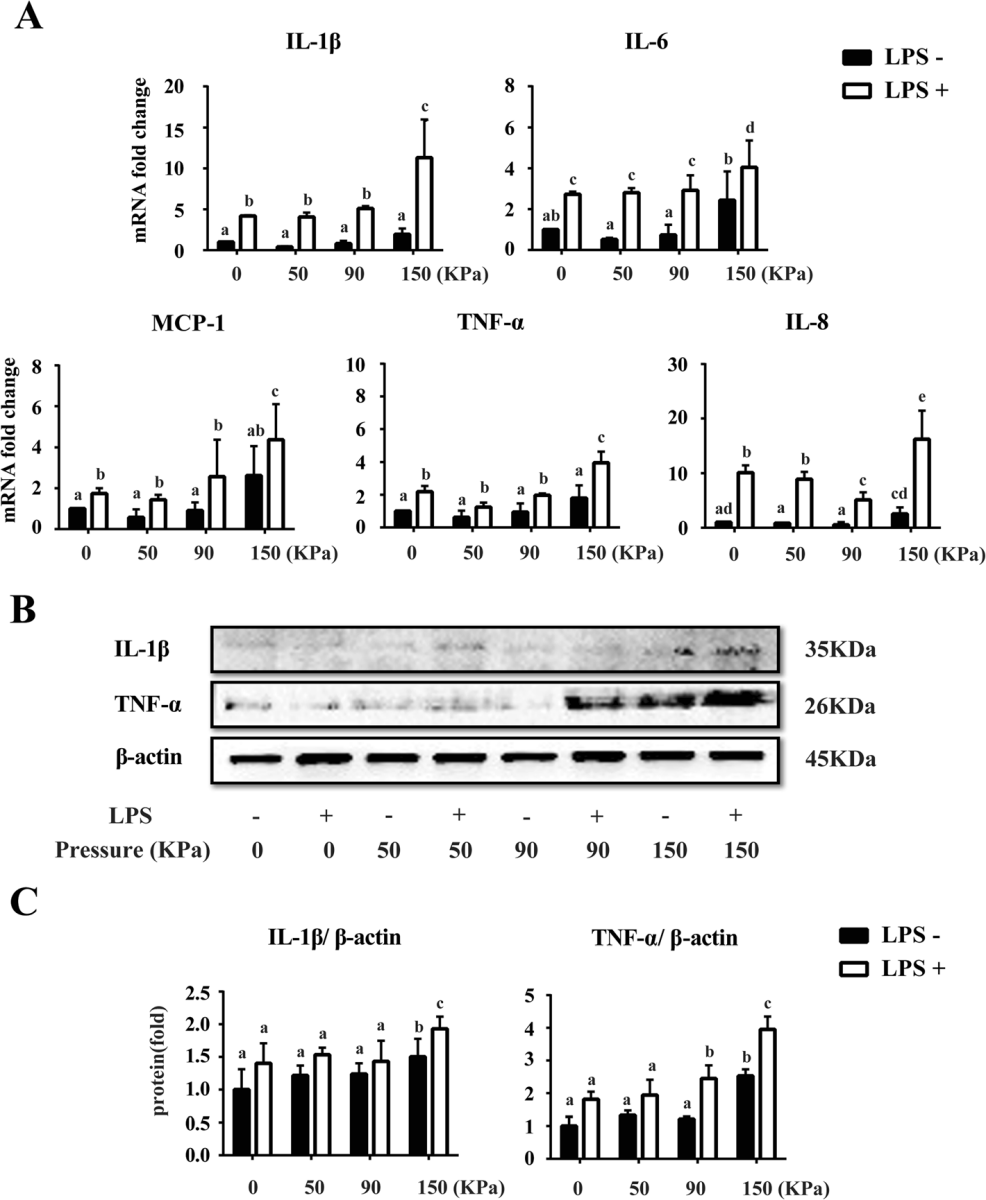
Expression of mRNA and protein levels of the inflammatory markers in hPDLCs after different dynamic cyclic stress loading for 5 days**. a** Real-time PCR results of pro-inflammatory markers, including *IL-1β*, *IL-6*, *IL-8*, *TNF-α* and *MCP-1* mRNA expression in hPDLCs after different cyclic stress loading for 5 days with LPS or not. **b** Western blotting analysis for IL-1β and TNF-α using total protein isolated from different groups of hPDLCs. **c** Quantification of Western blotting analysis. Protein content was expressed relative to the control and represented three similar independent experiments with triplicate observations in each experiment. Data were represented as the means ± SEM, n = 6 (hPDLCs from six donors). The bars with different lowercase letters were significantly different from each other (*P* < 0.05), and those with the same letter exhibited no significant difference

Given that 10 μg/ml LPS would induce the inflammation model, we then investigated the inflammation status of the hPDLCs under both 10 μg/ml LPS and different dynamic cyclic stress. From the results of real-time PCR, among the different loading groups of dynamic cyclic stress, all the LPS(+) groups showed significant higher mRNA expression of the pro-inflammatory cytokines including *IL-1β*, *IL-6*, *IL-8*, *MCP-1* and *TNF-α* compared to the corresponding loading groups of LPS(−) (*P* < 0.05). LPS(+)/0–150 kPa dynamic cyclic stress loading treatment up-regulated all the pro-inflammatory cytokines (*P* < 0.05), while LPS(+)/0–50 kPa and LPS(+)/0–90 kPa treatment showed no significant effect on the expressions of those cytokines, compared to LPS(+)/0 kPa group. Among the LPS(−) groups, the mRNA expression of *IL-1β*, *MCP-1* and *TNF-α* showed an increasing tendency as the loading increased, which came to a head in the LPS(−)/0–150 kPa group. In addition, LPS(−)/0–150 kPa loading significantly up-regulated the expression of *IL-6* and *IL-8* compared with both the LPS(−)/0–50 kPa and LPS(−)/0–90 kPa groups (*P* < 0.05) (Fig. 2a).

Since IL-1β and TNF-α are two of the most important inflammatory factors in the progress of periodontitis, we characterized the effects of different dynamic cyclic stress on these protein expressions in LPS-induced inflammatory hPDLCs to extend our observations at the mRNA level. Consistent with the gene expression pattern, Western blotting analysis demonstrated that after treated with LPS, the expression level of proteins IL-1β and TNF-α in hPDLCs showed obvious enhancement no matter which range of the cyclic stress was loaded. Especially compared to the LPS (−)/0–90 kPa group, the LPS(+)/0–90 kPa group exhibited significant more expression of TNF-α protein. Meanwhile, both IL-1β and TNF-α expressed higher in the 0–150 kPa group than the other loading groups, no matter the hPDLCs was treated with or without LPS. What’s more, the LPS(+)/0–150 kPa treatment induced the highest expression of IL-1β and TNF-α protein than all the other groups, which showed the exacerbation of inflammatory status after over loading on the LPS-induced hPDLCs (Fig. 2b and c).

### The osteoblastic effects of different dynamic cyclic stress on LPS-induced inflammatory hPDLCs

**Fig. 3:**
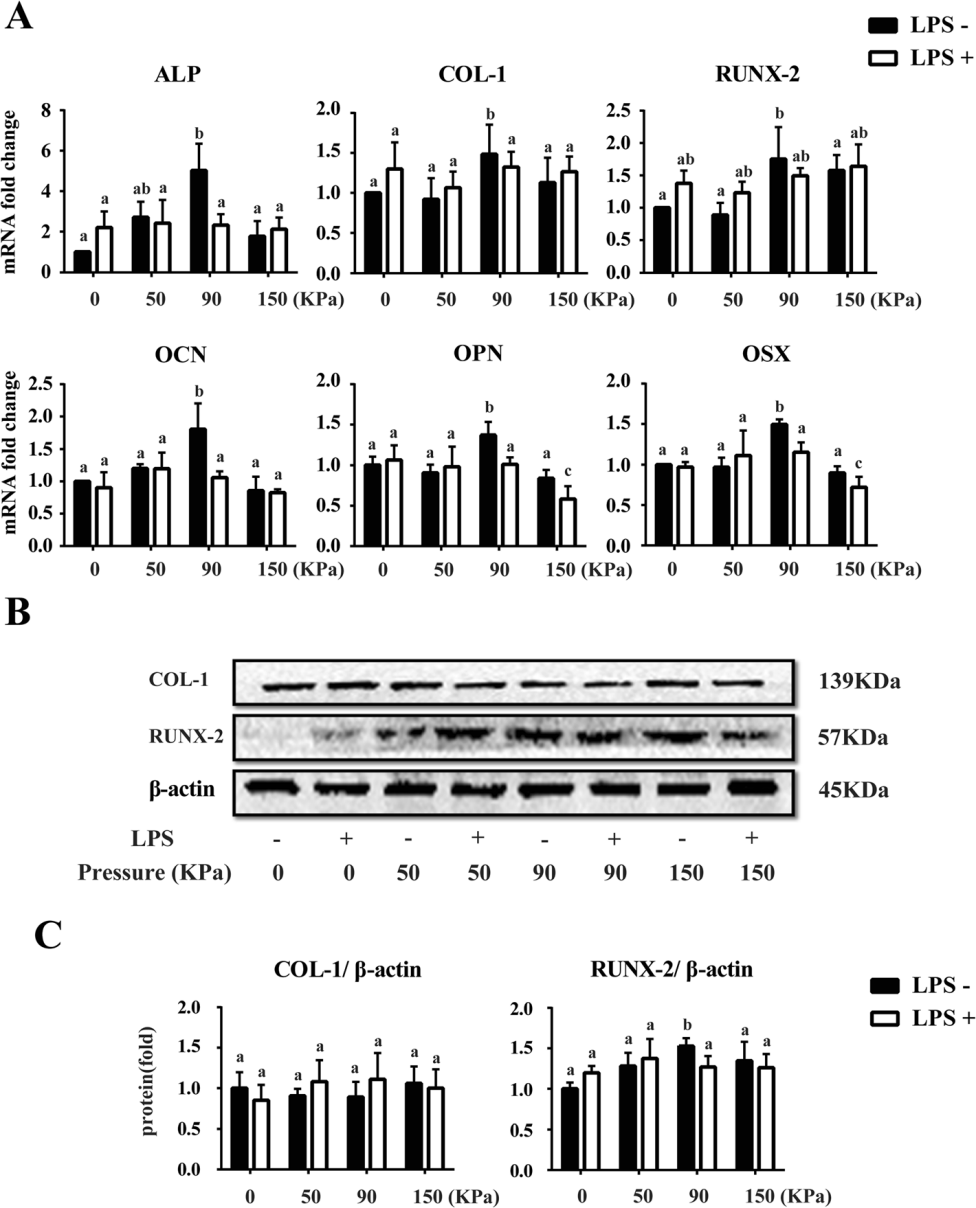
Expression of mRNA and protein levels of the osteoblastic markers in hPDLCs after different dynamic cyclic stress loading for 5 days**. a** Real-time PCR results of osteoblastic markers *ALP*, *COL-1*, *RUNX-2*, *OCN*, *OPN* and *OSX* mRNA expression in hPDLCs after different cyclic stress loading for 5 days with LPS or not. **b** Western blotting analysis for COL-1 and RUNX-2 using total protein isolated from different groups of hPDLCs. **c** Quantification of Western blotting analysis. Protein content was expressed relative to the control and represented three similar independent experiments with triplicate observations in each experiment. Data were represented as means ± SEM, n = 6 (hPDLCs from six donors). The bars with different lowercase letters were significantly different from each other (*P* < 0.05), and those with the same letter exhibited no significant difference

Among the four loading groups without LPS treatment, after 5 days of 0–90 kPa dynamic cyclic stress loading, the mRNA expressions of osteoblastic cytokines alkaline phosphatase (*ALP*), *COL-1* and osteocalcin (*OCN*) were up-regulated to the greatest extent, compared with the 0 and 0–50 kPa groups. Then they declined to the basal line after 5 days of 0–150 kPa dynamic cyclic stress loading. Meanwhile, the mRNA expression of *RUNX-2* in LPS(−)/0–150 kPa group was also promoted as with the LPS(−)/0–90 kPa group, compared with the LPS(−)/0 kPa and LPS(−)/0–50 kPa groups. However, there was no significant difference in any expression of the osteoblastic cytokines between LPS(−)/0 kPa and LPS(−)/0–50 kPa groups. Similar to the expression pattern of *ALP*, *COL-1*, *OCN* and *RUNX-2*, osteopontin (*OPN*) and osterix (*OSX*) also showed significant higher mRNA level in the LPS(−)/0–90 kPa group than the other LPS(−) groups. What’s more, the expression of these two osteogenic related genes decreased distinctly in the LPS(+)/0–150 kPa group among all the other groups and reached a statistical significance. But no significant difference among the other LPS(+) groups after the different loadings was found (Fig. 3a).

In accordance with the results of real-time PCR, Western blotting analysis revealed that the expression level of RUNX-2 protein reached the peak at LPS(−)/0–90 kPa group compared with the other LPS(−) loading groups. However, the RUNX-2 protein level showed no significant difference among the different loading groups in LPS-induced hPDLCs. Moreover, the expression of COL-1 protein had no obvious change among the different dynamic cyclic stress groups no matter treated with LPS or not after 5 days (Fig. 3b and c).

### The osteoclastic effects of different dynamic cyclic stress on LPS-induced inflammatory hPDLCs

**Fig. 4:**
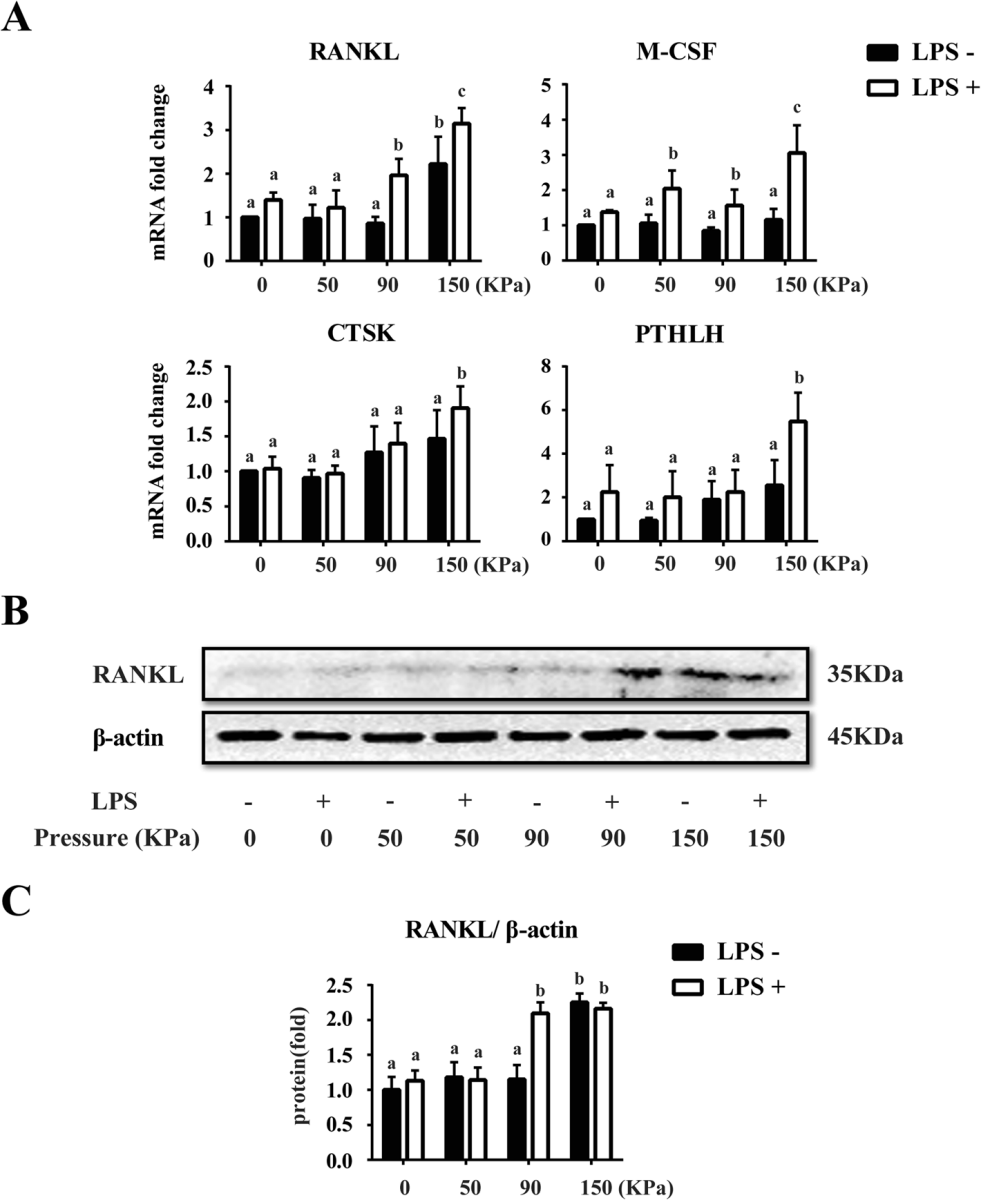
Expression of mRNA and protein levels of the pro-osteoclastic markers in hPDLCs after different dynamic cyclic stress loading for 5 days. **a** Real-time PCR results of pro-osteoclastic markers *RANKL*, *M-CSF*, *CTSK* and *PTHLH* mRNA expression in hPDLCs after different cyclic stress loading for 5 days with LPS or not. **b** Western blotting analysis for RANKL using total protein isolated from different groups of hPDLCs. **c** Quantification of Western blotting analysis. Protein content was expressed relative to the control and represented three similar independent experiments with triplicate observations in each experiment. Data were represented as means ± SEM, n = 6 (hPDLCs from six donors). The bars with different lowercase letters were significantly different from each other (*P* < 0.05), and those with the same letter exhibited no significant difference

Having observed the changes in osteoblastic cytokines after the treatment of different dynamic cyclic stress and LPS, we then investigated the expression changes of the pro-osteoclastic cytokines. RT-PCR results suggested that the mRNA levels of pro-osteoclastic cytokines, including *RANKL*, macrophage colony-stimulating factor (*M-CSF*), *CTSK*, *PTHLH* in LPS-induced hPDLCs were up-regulated compared to the corresponding loading groups without LPS respectively, and expressed the statistical difference at the 0–150 kPa group. Among four LPS(−) groups, it could be seen that the mRNA level of pro-osteoclastic cytokines went up following the loads increased. Similar to the LPS(−) groups, the expression of these osteoclastic markers was significantly promoted in the LPS(+)/0-90 kPa groups, and reached the highest level in the LPS(+)/0–150 kPa group after 5 days (Fig. 4a).

The result of Western blotting showed that LPS(+)/0–90 kPa and LPS(+)/0–150 kPa dynamic cyclic stress treatment could up-regulate the expression level of RANKL protein, compared with other LPS(+) groups. In addition, only 0–150 kPa dynamic cyclic stress treatment without LPS could also promote the expression of RANKL protein to an extreme high level than the other LPS (−) groups. There was no obvious effect of osteoclasts after the cyclic stress under 90 kPa regardless the LPS treatment or not. Nevertheless, the synergistic effect of a smaller cyclic stress and LPS treatment on promoting the osteoclasis in hPDLCs was nearly equal to the much more dynamic cyclic stress treatment without LPS (Fig. 4b and c).

## Discussion

As known, the process of periodontitis begins with the endotoxin released by bacteria. Then the pro-inflammatory factors such as TNF-α and IL-1β are secreted by local periodontal ligament cells, which invades periodontal tissues and finally leads to the absorption and destruction of parodontium [16]. TNF-α and IL-1β were demonstrated to be the key factors in periodontits [17]. From another perspective, LPS, by which endotoxin exists on the walls of some bacteria, can lead to inflammatory reactions in multiple tissues such as genitourinary inflammation [18] and chronic fatigue syndrome [19]. It was reported that LPS can also stimulate the defensive cells in PDL to produce pro-inflammatory factors such as TNF-α and IL-1β, and then cause the destruction of PDL and alveolar bone [1, 20]. However, the concentration of LPS applied on hPDLCs to mimic the inflammation in vitro is still controversial. 0.1–10 μg/ml LPS were used on hPDLCs to induce the inflammatory state to observe the effects of *IL-6* and *MCP-1* in the previous study [21]. And it was also reported that 1.0 μg/ml of LPS could be used to establish the inflammation model and could contribute to the secretion of inflammatory cytokines in hPDLCs [22]. The miRNA expression patterns were investigated in the inflammatory hPDLCs induced by 0, 0.5, 1.0, 1.5 and 2.0 μg/ml of LPS [23]. What’s more, the scholars also applied 0, 10, 20, 50, and 100 μg/ml of LPS on hPDLCs to make an inflammatory environment, and aimed to investigate the anti-inflammatory effect of a certain therapy [24]. So, we selected the concentrations of 0.1, 1.0, 10, 100 and 500 μg/ml to make clear the appropriate working dosage of LPS in the inflammation induction of hPDLCs in vitro. According to both MTT assay and real-time PCR analysis, it could be concluded that 10 μg/ml of LPS showed no effect on the proliferation of cells and promoted the inflammatory response of hPDLCs, which could be used to induce the model of inflammation in vitro in the following study.

Mechanical stress is essential for the physiological function of a healthy periodontium. But excessive occlusal stress could cause the damage of periodontal tissue in vivo. Cyclic hydrostatic pressure has been applied on hPDLCs in vitro to mimic the physiological state of periodontium [15]. Previous studies had suggested that cyclic pressure higher than 150 kPa could significantly affect the morphology and function of hPDLCs and also inhibit the proliferation and differentiation of these cells. What’s more, it had been reported that the feasible pressure condition for hPDLCs should be 90 kPa for 60 min, under which the ALP activity of the cells would be promoted without affecting cell proliferation rates [15]. Therefore, in our study, 0–50 kPa, 0–90 kPa and 0–150 kPa were selected as the loading range to simulate the state of normal occlusion, critical occlusion and over occlusion in vivo. And the conclusions of this study were accompanied with the previous finding that over loading of pressure would significantly upregulate the expression of the inflammation and osteoclast related markers on both healthy and LPS-induced inflammatory hPDLCs, which indicated that over loading may induce and enhance the inflammation of hPDLCs.

It is well known that PDLCs can be induced to differentiate into osteoblasts and pre-osteoclasts, and participate in the bone remodeling according to different mechanical stimulations. In some orthodontic studies, new bone formation was always found on the tension side while the osteoclasts on the pressure side were extremely active [25]. Studies showed PDLCs under cyclic stretch stress could express higher OCN, ALP and Runx2 [26]. While there were few researches about the compression-relative osteoblastic differentiation. It was reported that the expression of osteogenic factors in MC3T3 E1 cells increased significantly after the cyclic pressure applied within a certain range [27]. Therefore, we speculated that appropriate cyclic pressure may promote the osteogenic differentiation in hPDLCs. Combined with the researches that RUNX2 also associated with cell proliferation and could not be used to indicated the role on osteogenic differentiation by itself [28, 29]. In the present study, we also detect the expression level of *OCN*, *OPN* and *OSX*, who were regarded as the markers in the maturity of osteoblast and bone mineralization to verify the osteogenic differentiation of hPDLCs after dynamic cyclic stress during LPS-induced inflammation [30, 31]. Through real-time PCR detection, we found an obvious up-regulated expression of *ALP*, *COL-1*, *RUNX2, OCN*, *OPN* and *OSX* in healthy hPDLCs under the dynamic cyclic pressure of 0–90 kPa, which suggested this certain range of stress could promote osteogenesis-related gene expression, including both early-stage genes and advanced-stage genes. In addition, both OPN and OSX mRNA level showed a significant decrease after 5 days of 0–150 kPa cyclic pressure in a LPS-induced inflammatory environment among the other corresponding LPS(+) groups. But there was no similar observation in the Western blotting analysis among these groups. The reason for this inconsistency may be related to the short experimental period and the lack of osteogenic induction medium during the experiment, which still warrants further investigation. These results may indicate that appropriate mechanical stimulation could promote osteogenic differentiation of hPDLCs and is the favorable factor for the bone remodeling in the healthy periodontium, whereas this positive effect would be interfered by inflammation.

Otherwise, we found that the expression pattern of pro-osteoclastic cytokines was similar to the pro-inflammatory cytokines among the groups in this present study. The results of this study showed the effect of osteoclasts after over loading of cyclic stress on the healthy hPDLCs without LPS, indicating that over loading could also induce the bone resorption in healthy periodontium. In addition, after dynamic cyclic pressure and LPS treatment, the osteoclastic and inflammatory effects on the hPDLCs were both aggravated. In our present study, both mRNA and protein expression level of RANKL increased significantly after the LPS plus 0–90 kPa or 0–150 kPa dynamic cyclic pressure treatment. And the mRNA expression of CTSK and PTHLH were also upregulated in the LPS(+)/0–150 kPa group. RANKL is an important pre-osteoclastic marker, appearing to be both necessary and sufficient for the complete differentiation of osteoclast pre-cursor cells into mature osteoclasts. Previous studies showed that the expression of RANKL can be up-regulated under compressive force in PDLCs, which is an essential factor for osteoclastogenesis [32]. M-CSF has been shown to be an essential factor in addition to RANKL for the differentiation and the survival of osteoclasts [33]. In this study, we provided evidence that LPS(+)/0–150 kPa increased the mRNA expression of M-CSF, which consisted with the expression pattern of other osteoclastic markers. Moreover, except for strongly expressing by osteoclasts and specifically induced during osteoclast differentiation in general, CTSK was also found to contribute to the destruction of bone and the PDL in periodontitis based on its role in collagen degradation [34–36]. Interestingly, other studies showed that CTSK might have functions in the immune system of PDL cells besides its role in osteoclasts [37, 38]. Based on the present study, the conditions of osteoclastogenesis may be defined, which are over loading on healthy hPDLCs and the critical loading on LPS-induced inflammatory hPDLCs in vitro.

Studies have shown that cells in the periodontium are load sensitive, and mechanical stimulation regulate their functions, including proliferation, differentiation, and cytokine secretion, including the inflammatory cytokine [39]. As a result, the inflammatory response induced by LPS is aggravated by excessive mechanical stress, and the periodontal damage leads to the intolerance of mechanical stress further [40]. Furthermore, both excessive compressive force and inflammation would induce the hypoxic microenvironment which may take part in initiating osteoclastogenesis and may have combinatory effects with each other [10, 41]. It could update our concepts of the mechanisms involved in the osteoclast differentiation under mechanical stress and inflammation. However, the mechanism involved in the dual effect of cyclic compression forces on hPDLCs under LPS treatment still warrants further investigation.

## Conclusions

Therefore, we conclude that dynamic cyclic pressure can promote the osteogenic differentiation of healthy periodontal ligament cells in the physiological range of force (under 90 kPa). Within this certain range of force, the mechanical effects between osteogenesis and osteoclastogenesis on the inflammatory hPDLCs may have no significant difference from that of the healthy cells. But the excessive pressure (150 kPa) can significantly increase the release of inflammatory and pro-osteoclastic factors and decrease the expression of the osteogenic related genes, which could be further aggravated by LPS. This may suggest that the excessive occlusal force on periodontitis teeth can significantly aggravate the destruction of periodontal tissue and promote the progress of periodontitis ulteriorly. To sum up, according to the present study, 90 kPa could be a reference value to simulate natural occlusal force and 150 kPa may be an extreme loading for hPDLCs in vitro in the following biomechanical studies. However, the regulatory mechanism of the interactive effects between LPS and compression on the osteogenic differentiation of the hPDLCs is still unclear. And the subsequent animal experiments are also needed to confirm the conclusions of this study in the future.

## Data Availability

The datasets used and/or analyzed during the current study available from the corresponding author on reasonable request.

## Abbreviations

ALP: Alkaline phosphatase
BCA: Bicinchoninic acid
COL1: Type I collagen
DMSO: Dimethyl sulfoxide
hPDLCs: Human periodontal ligament cells
hPDLSCs: Human periodontal ligament stem cells
HRP: Horseradish peroxidase
IL-6: Interleukin-6
LPS: Lipopolysaccharides
MCP-1: Monocyte chemotactic protein 1
M-CSF: Macrophage colony-stimulating factor
MMPs: Matrix metalloproteinases
MTT: 3-(4,5-dimethylthiazol-2-yl)-2,5-diphenyltetrazolium bromide
OCN: Osteocalcin
OPN: Osteopontin
OSX: Osterix
PDL: Periodontal ligament
PTHrP: Parathyroid hormone-related protein
PVDF: Polyvinylidene difluoride
qRT-PCR: Quantitative real-time polymerase chain reaction
RUNX2: Runt-related transcription factor 2
TNF-α: Tumor necrosis factor-α
α-MEM: Alpha minimum Eagle’s medium
